# Evaluation of Surface Cleaning Procedures for CTGS Substrates for SAW Technology with XPS

**DOI:** 10.3390/ma10121373

**Published:** 2017-11-30

**Authors:** Erik Brachmann, Marietta Seifert, Steffen Oswald, Siegfried B. Menzel, Thomas Gemming

**Affiliations:** IFW Dresden, SAWLab Saxony, Helmholzstr. 20, 01069 Dresden, Germany; marietta.seifert@ifw-dresden.de (M.S.); s.oswald@ifw-dresden.de (S.O.); s.menzel@ifw-dresden.de (S.B.M.); t.gemming@ifw-dresden.de (T.G.)

**Keywords:** surface contamination, cleaning procedures, UV-ozone cleaning, RCA clean, CTGS, XPS

## Abstract

A highly efficient and reproducible cleaning procedure of piezoelectric substrates is essential in surface acoustic waves (SAW) technology to fabricate high-quality SAW devices, especially for new applications such SAW sensors wherein new materials for piezoelectric substrates and interdigital transducers are used. Therefore, the development and critical evaluation of cleaning procedures for each material system that is under consideration becomes crucial. Contaminants like particles or the presence of organic/inorganic material on the substrate can dramatically influence and alter the properties of the thin film substrate composite, such as wettability, film adhesion, film texture, and so on. In this article, focus is given to different cleaning processes like SC-1 and SC-2, UV-ozone treatment, as well as cleaning by first-contact polymer Opticlean, which are applied for removal of contaminants from the piezoelectric substrate Ca${}_{3}$TaGa${}_{3}$Si${}_{2}$O${}_{14}$. By means of X-ray photoelectron spectroscopy, the presence of the most critical contaminants such as carbon, sodium, and iron removed through different cleaning procedures were studied and significant differences were observed between the outcomes of these procedures. Based on these results, a two-step cleaning process, combining SC-1 at a reduced temperature at 30 ${}^{\circ }$C instead of 80 ${}^{\circ }$C and a subsequent UV-ozone cleaning directly prior to deposition of the metallization, is suggested to achieve the lowest residual contamination level.

## 1. Introduction

For the preparation of high-quality thin films on substrates a clean surface of the substrates is indispensable. Contamination can occur in the form of particles, closed films, or adsorbed gases and might adversely affect the film growth or lead to a reduced adhesion strength of the deposited film or result in undesired chemical reactions with either the film or the substrate material. A short overview on the impact of different contaminations was summarized by Kohli and Mittal in the introduction of their book [1]. Leopold distinguishes between molecular, ionic, and atomic sources of contaminations [2]. To remove contaminants, different cleaning procedures of the substrates prior to the film deposition are generally applied. There are some very effective cleaning procedures for silicon, which are well established [3] and have been optimized over several decades [2]. The standard cleaning procedure for silicon substrates in microelectronics is the so-called RCA-procedure (see Section 2.3) named after the Radio Cooperation of America and developed by Kern and Puotinen in 1970 [4]. Other cleaning processes were also designed, like UV-ozone treatment, developed by Vig in 1985 [5]. However, the drawback with this method is the thickening of oxide layer on a silicon surface that occurs [6]. Another common method is oxygen plasma cleaning [7]. A comparison between UV-ozone and oxygen plasma cleaning processes was described by Choi et al. [8].

However, during the cleaning process itself, new contaminants can be introduced, for example, from cleaning equipment, impurities of used chemicals, and ambient conditions. Therefore, careful handling of these devices and chemicals is imperative to maintain them in a clean state.

Adequate information and knowledge is available about the cleaning of Si/SiO${}_{2}$ substrates. However, there are many advanced applications that need removal of specific substrates with specific properties for which the information that exists is barely adequate. One example is the high-temperature sensor devices which are based on surface acoustic waves (SAW) that require the use of high-temperature-stable piezoelectric substrates. One of the most promising candidates is Ca${}_{3}$TaGa${}_{3}$Si${}_{2}$O${}_{14}$ (CTGS). The piezoelectric properties of CTGS and its suitability as a substrate for SAW applications have been investigated by several groups [9,10,11]. It is possible to excite this material piezoelectrically up to at least 1285 ${}^{\circ }$C, which is very close to its melting temperature of 1350 ${}^{\circ }$C [12]. High-temperature-stable metallizations for interdigital transducers on CTGS based on, for example, intermetallic or refractory materials, were studied by Seifert et al. [13] and also by Rane et al. [14].

Compared to Si, CTGS has a different surface chemistry and, hence, has a different affinity to common contaminants. As such, a well-established cleaning procedure will yield different results when compared to Si. Thus, it becomes important to develop a cleaning procedure that can remove contaminants as thoroughly as possible as well as produce the same surface conditions before the film deposition, so as to ensure reproducibility. Therefore, a detailed analysis of the surface of both substrates is the focus of this article.

Six cleaning procedures applied to CTGS substrates are compared in reference to Si substrates. For this purpose, X-ray photoelectron spectroscopy (XPS) was used to determine the residual contamination after the cleaning processes and to quantify the efficiency of cleaning.

XPS is an ideal tool for monitoring surface contamination of solid samples [15]. Besides the detection of any element (except hydrogen) by means of specific electron energies, XPS can also identify characteristic chemical bonds based on peak shifts. The high surface sensitivity (information depth below 5 nm) allows the detection of surface contamination monolayers as well. Another advantage of XPS is it is possible to easily investigate electrically insulating samples like CTGS substrate, since no charged particles are used for excitation. The investigation of insulating materials is challenging with many other methods that use charged particles, for example, Auger electron spectroscopy.

## 2. Materials, Cleaning Processes, and Analytical Methods

The substrates (7 mm × 8 mm) were prepared by sawing them from CTGS Y-cut wafers (diameter 75 mm, Fomos Materials, Moscow, Russia). As a reference 10 mm × 10 mm, boron-doped Si (100) substrate with 1000 nm thermal oxide was used (Silicon Materials, Kaufering, Germany). Before sawing with a commercial wafer dicing saw (DAD3220, Disco Inc., Bunkyo-ku, Tokyo, Japan), substrates were covered with a protection layer of photoresist type AZ-520 D (AZ Electronic Materials GmbH, Darmstadt, Germany) by spin-coating for 2 min at 3000 rpm. After the resist dried, the wafers were cut in 7 mm × 8 mm or 10 mm × 10 mm sized pieces. During the dicing distilled water with an electrical conductivity of 0.5-5 μS/cm was used to rinse the wafers to cool the wafer saw and to remove sawdust. The residual protection layer was removed by cleaning the substrates with the dimethylsulfoxide (DMSO)-cleaning procedure which is described in Section 2.2. The prepared samples were stored in a moisture-free box until the cleaning procedures were carried out. The cleaning procedures explained in the following sections were carried out in a HERA safe box (KS 12, Thermo Fischer Scientific GmbH, Waltham, MA, USA) to reduce particle contamination from ambient air during cleaning. All of the beakers that were used during the cleaning process were made of quartz glass to reduce contamination of ultra-pure water (electrical conductivity below 0.05 μS/cm) with silicic acids that can dissolve out from standard glass beaker materials made of borosilicate glass. All the solvents used in the cleaning processes possessed analytical quality. The ultra-pure water was obtained by a special water-conditioning device (Reinstwasseranlage, Weinert Wasseraufbereitung GmbH, Dresden, Germany) which purifies water and has the following parts: water softener, ion exchanger, reverse osmosis plant, UV-light irradiation unit, synthetic resin ion exchanger, and activated carbon filter. This water was used for rinsing between individual cleaning steps and for final rinsing in the ultrasonic bath. Every rinsing process of the samples is accomplished in three steps, including the so-called cascaded rinsing. At the end of the cleaning procedure, the substrates were blow-dried using dry nitrogen gas (purity of 99.999%) for about 10 s with a gas pressure of less than 1 bar. In the following sections, the different cleaning procedures investigated are explained in detail:

### 2.1. Cleaning in Acetone-Ethanol-Mixture

The use of both acetone and ethanol for cleaning samples is common in laboratory settings, since both solvents are commonly available in laboratories and are quite easy to handle compared to use of acids in the RCA procedure. Therefore, this cleaning method is included in our investigations to study its suitability for removal of contaminants. Menon et al. developed a cleaning procedure for removing inorganic particles using a mixture of acetone and ethanol (volume ratio 1:1) in an ultrasonic bath for 15 min at room temperature [16]. After this cleaning step, the sample was rinsed twice with ultrapure water followed by an ultrasonic bath for 15 min.

### 2.2. Cleaning in Dimethylsulfoxide (DMSO)

In photolithography laboratories, DMSO is often used because of the upcoming need to use less harmful solvents. There are no hazard and precautionary statements for DMSO in contrast to the regulations imposed on the use of acetone and ethanol. Another advantage is that DMSO allows cleaning at higher temperatures because of its higher boiling point as compared to acetone and ethanol. The cleaning process takes place within temperatures ranging between 70 and 75 ${}^{\circ }$C in an ultrasonic bath for 15 min. The samples are then flushed with isopropanol, followed by further cleaning by rinsing the samples in isopropanol in an ultrasonic bath for 15 min at room temperature.

### 2.3. RCA Clean Procedure

The RCA cleaning process is an oxidizing and complex-forming two-step cleaning procedure that uses hydrogen peroxide. The first step, the so-called RCA standard clean 1 (SC-1), is done in an alkaline mixture with a high pH value. The mixture consists of ammonia (29 vol % NH${}_{3}$), hydrogen peroxide (30 vol % H${}_{2}$O${}_{2}$), and water (ultrapure < 0.05 μS/cm) in the ratio of 1:1:5 (NH${}_{3}$:H${}_{2}$O${}_{2}$:H${}_{2}$O) [4] at temperatures ranging from 75 to 80 ${}^{\circ }$C. The amount of additives in the hydrogen peroxide should be as low as possible to reduce recontamination of the surface [2]. SC-1 leads to a significant reduction of the organic contaminants on the sample surface and, additionally, to dissolution of metals of groups IB and IIB and elements like Au, Ag, Cu, Ni, Cd, Zn, Co, and Cr by forming dissolvable complexes [2]. The second step, the so-called RCA standard clean 2 (SC-2), is done in an acidic solution with low pH value. The solution consists of hydrochloric acid (37 vol % HCL), hydrogen peroxide (30 vol % H${}_{2}$O${}_{2}$), and water (ultrapure < 0.05 μS/cm) in the ratio of 1:1:6 (HCl:H${}_{2}$O${}_{2}$:H${}_{2}$O) [4] at temperatures ranging from 75 to 80 ${}^{\circ }$C. The main purpose of this second cleaning step is to remove alkali metals (group IA) and their ions and other metallic particles, which could not be removed during the SC-1 cleaning step, for example, Al, Fe, and Mg. Here, recontamination of the surface is prevented due to the formation of soluble chemical complexes and the passivation effect of hydrochloric acid [2]. After the conclusion of SC-2 step, rinsing of the samples with ultrapure water and further rinsing in an ultrasonic bath for 15 min at room temperature are carried out.

### 2.4. Modified RCA-Cleaning Procedure: SC-1 at Room Temperature

It is common practice in the industry, for the simplification of RCA cleaning procedure, to do SC-1 cleaning at a reduced temperature of 30 ${}^{\circ }$C. This simplified process is attractive because of the easier compliance of industrial health and safety standards. The decrease of temperature is also attractive for laboratory use due to the easier handling.

### 2.5. Cleaning in UV-Ozone Atmosphere

UV-ozone cleaning based on results of Vig [5] is carried out in a closed chamber (UVOH 150 LAB, FHR Anlagenbau GmbH, Ottendorf-Okrilla, Germany). The cleaning process starts with flushing the chamber with nitrogen gas for 2 min and subsequent filling with oxygen (O${}_{2}$ flow rate: 1 l/min). By setting a process time, in our case 10 min, the UV lamp is activated which emits ultraviolet light with characteristic wavelengths of 184.9 nm and 253.7 nm to form the ozone. The high reactivity of ozone leads to cracking of hydrocarbon bonds at the substrate surface and formation of gaseous CO${}_{x}$. After cleaning, the chamber is again flushed with nitrogen for 2 min to remove the residual ozone in the chamber.

### 2.6. Cleaning by Use of Opticlean First-Contact Polymer

First-contact polymers are used in optics or optical microscopy for cleaning the eyepiece or ocular lenses. In SAW technology, especially for applications in SAW-driven microacoustics, these polymers are often used to cover IDT structures or to remove particles from the surface. The liquid polymer is deposited with a soft brush directly onto the surface. After drying out the solvents for approximately 10–15 min a film is formed that can be removed by pulling it with a tweezer.

For this article the different cleaning procedures were organized in such a way that they were completed almost at the same time to reduce time differences of the exposure of the samples to ambient conditions. After completing the cleaning procedures, six samples were placed on a sample holder. Then the holder was protected with a glass cover and was directly (less than 5 min after the cleaning process) transferred to the XPS system. The XPS measurements were carried out on a PHI 5600 CI (Physical Electronics, Chanhassen, MN, USA) spectrometer which is equipped with a hemispherical analyzer operated at a typical pass energy of 29 eV and 800 μm diameter analysis area defined by the entrance lens system of the analyzer. The base pressure in the XPS chamber is typically less than $2\cdot 10^{-8}$ Pa. Non-monochromatized Al Kα excitation (400 W) was used because the normally used Mg Kα excitation causes an overlap of the C1s peak characteristic with an LMM Auger line of Ga which is present in the CTGS substrate. The take-off angle of the analyzer is 45${}^{\circ }$; the X-ray source is positioned to the sample surface in the “magic-angle” of 54.7${}^{\circ }$ for minimizing the asymmetry effects. The energy scale of the spectrometer was calibrated to Au4f${}_{7/2}$ at 84.0 eV and Cu2p${}_{3/2}$ at 932.65 eV, with an accuracy of 0.05 eV. However, the routinely achievable long-term stability of the spectrometer is specified as ±0.1 eV. The energy resolution reached for the Al X-ray source results in a full-width-at-half-maximum (FWHM) measured with 29 eV pass energy at Ag3d${}_{5/2}$ of 1.1 eV. Due to the insulating character of the substrate material an additional low-energy electron charge neutralizer (5 eV, approx. 300 nA) was applied. For concentration quantification with the PHI Multipak Software [17], standard single-element sensitivity factors were used which are based on data collected by previous studies [18] and by considering the actual analyzer transmission performance. The considered photoelectron peaks were: C1s, O1s, Ca2p, Ta4f, Ga2p${}_{3/2}$, Si2s, Fe2p${}_{3/2}$, and Na1s. The peak intensities are determined by using Shirley-background subtraction. In addition, a homogeneous element distribution is assumed within the information depth of the XPS method. Considering the attenuation length (AL) for the measured electrons in CTGS of about 2 nm and the fact that 95% of the signal is coming from the depth of 3 × AL this can be assumed to be around 6 nm. The concentration values are calculated with the simple concept of relative-sensitivity-factors not including further matrix or AL corrections [18].

This has to be taken into consideration when evaluating the calculated results because the surface species are at least overestimated with respect to substrate elements. However, a comparison between different sample states when measured under identical conditions is always possible. For the measuring conditions used, the detection limit for the considered elements can be determined from the signal-to-noise ratio to be approximately 0.1 at %. The measurements show, besides the elements of the substrate, exclusively peaks caused by carbon, sodium, and iron. The peak shape/position at 710 eV of the Fe2p${}_{3/2}$ peak point to iron oxide/hydroxide [18], whereas the exact state cannot be determined because of the low signal-to-noise ratio. The Na1s found at 1072 eV is not so sensitive to chemical surrounding [18] but can be also assumed as oxide/hydroxide because other potential binding partners (e.g., Cl) could not be observed. However, the comparison of methods focuses on the amount of these contaminations while the exact chemical state is less important. The XPS results are evaluated so that the total amount of all present species is 100 at %. However, the graphs only demonstrate the atomic concentration of the respective element.

## 3. Results and Discussion

**Figure 1:**
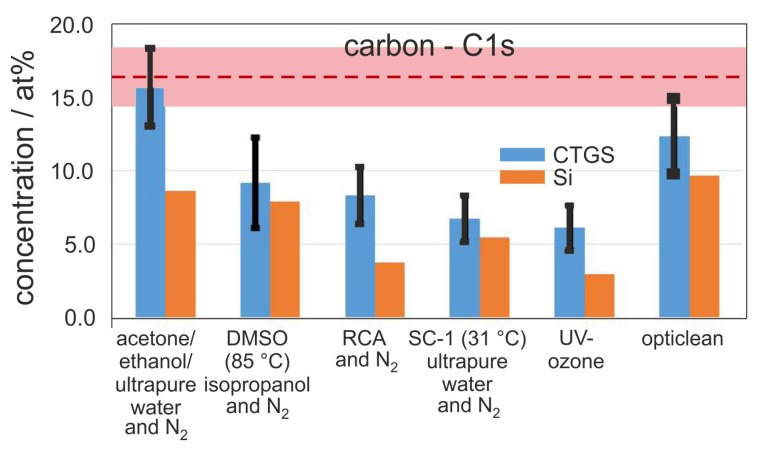
Mean value of carbon concentration from residual hydrocarbon surface contamination after the respective cleaning procedure measured with X-ray photoelectron spectroscopy (XPS) (Al-source with neutralizer) on the Ca${}_{3}$TaGa${}_{3}$Si${}_{2}$O${}_{14}$ (CTGS) surface and on silicon reference. The red dashed line shows the mean carbon value of the unclean CTGS reference samples of about 17 at % C. The light red stripe indicates the corresponding standard deviation of 2.0 at %.

**Figure 2:**
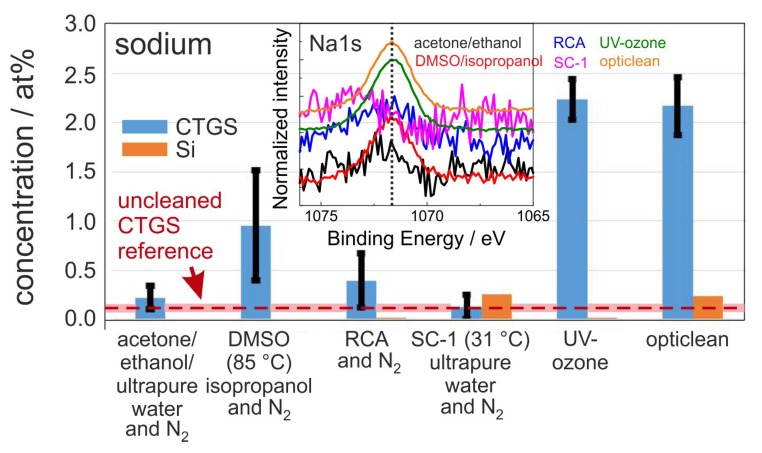
Mean value of sodium concentration after the respective cleaning procedure measured with XPS (Al-source with neutralizer) on the CTGS surface and on Si reference. The red dashed line represents the mean sodium values of the unclean CTGS reference samples of about 0.14 at %. The light red stripe indicates the corresponding standard deviation of 0.04 at %. The inset shows the normalized Na1s spectra of one set of samples. The black dotted line marks the theoretical position of the Na1s peak.

**Figure 3:**
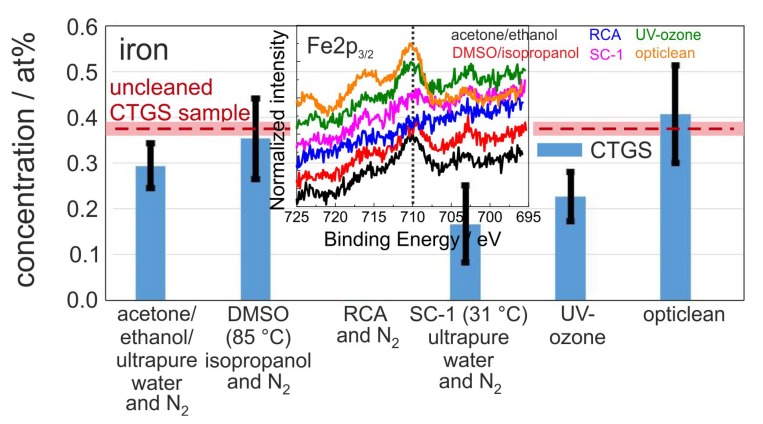
Mean value of iron concentration after the respective cleaning procedure measured with XPS (Al-source with neutralizer) on the CTGS surface. The red dashed line represents the mean iron values of the uncleaned CTGS reference samples of about 0.38 at %. The light red stripe indicates the corresponding standard deviation of 0.015 at %. For the Si reference, no Fe contamination was detected. The inset shows the normalized Fe2p${}_{3/2}$ spectra of one set of samples. The black dotted line marks the theoretical position of the Fe2p${}_{3/2}$ peak.

In the following section, the results of the XPS analysis for the three detected contamination elements C, Na, and Fe after the respective cleaning procedures are summarized together with the amount of contaminants for the as-supplied CTGS substrates. This reference value is marked in Figure 1, Figure 2 and Figure 3 with the dashed line. The reference samples have neither been cleaned nor have been in contact with any liquids. They have been obtained by breaking and not by sawing the wafer. The reported contamination level of these reference samples is a mean value of four individual samples. Since one aim of this work is to compare the results of the cleaning procedures on CTGS with those obtained on Si substrates, in each figure the results for Si are additionally presented.

In Figure 1, the mean carbon concentration from the residual hydrocarbon contamination at the substrate surface after applying the six different cleaning procedures is shown. Measurements were done for three repetitions which were independent of each other. The mean value is calculated from the three determined carbon values and presented together with the standard deviation. In the following section, we will distinguish between absolutely measured atomic concentrations “at %” and their relative changes “%”.

For all methods a reduction of the mean carbon content on the surface after the cleaning procedures is observed. The lowest decrease of carbon contamination of about 8% can be seen for the acetone/ethanol cleaning (16 at % residual carbon content, rcc), followed by the polymer cleaning procedure with a carbon reduction of 27% (12 at % rcc). The DMSO cleaning reduces 46% of the carbon (9 at % rcc), the RCA cleans 51% (8 at % rcc), and the reduced SC-1 cleans 60% (7 at % residual carbon content). The best results were achieved with the UV-ozone cleaning, which leads to a reduction of 64% and to only 6 at % residual carbon. Earlier XPS measurements reported by Vogel et al. showed comparable results for the mean carbon concentration on LiNbO${}_{3}$ substrates of 11 at % C and on LiTaO${}_{3}$ substrates of 15 at % for the SC-1 cleaning procedure [19]. However, the residual carbon concentration on the CTGS substrates is larger for all methods, in some cases up to a factor of 2, as compared to the cleaned Si substrates.

Figure 2 summarizes the mean sodium content. Sodium is an indicator for the cleaning impact on alkali metals and for salt contamination introduced during the cleaning process itself. Obviously, for nearly all procedures the Na content increased strongly as compared to the uncleaned reference. This difference between processed and non-processed samples might be explained by the residual content of salt in ultrapure water or in other solvents which condense during pre-processing, for example, during spin coating, cutting, protective lacquer coating, or removing of this before the actual cleaning procedure takes place.

Specifically, the sample cleaned by SC-1 procedure with reduced temperature (30 ${}^{\circ }$C) shows a reduction of sodium content. In contrast to this, the RCA cleaning with additional SC-2 cleaning step leads to a higher Na concentration. This difference might be caused by further cleaning steps and treatments also with water and possibly higher activation of substrate surface for the adsorption of Na. The highest value of about 2.2 at % Na was measured for the UV-ozone and the opticlean polymer process. In contrast to the CTGS substrates, the Si substrates seem to be much less sensitive to Na adsorption. Only in the case of reduced RCA and the opticlean method appreciable amounts of Na were detectable.

The normalized Na1s spectra of one set of samples are summarized in the inset of Figure 2. A clear peak is only visible for the DMSO, UV-ozone, and opticlean methods, and the others show only a very small peak which is mainly noise.

Figure 3 presents the mean iron concentration at the sample surfaces, which serves as an indicator for metallic contaminations. In contrast to CTGS substrates, which show, except for the RCA cleaning, a very small but verifiable Fe contamination before and after cleaning, for none of the Si samples the presence of Fe was proven. For CTGS only the RCA cleaning results in complete removal of the iron contamination which is visible from the pure noise signal of the Fe2p${}_{3/2}$ spectra for the RCA cleaning procedure in the inset in Figure 3. It appears that both the opticlean polymer and DMSO-cleaning procedures do not have any effect on iron contaminations on the surface. The acetone-ethanol-mixture cleaning leads to a slight reduction in iron content, and, also, surprisingly, the UV-ozone cleaning slightly reduces the Fe content.

In addition to the immediate cleaning effect of the different methods the accumulation of carbon contamination over a certain time is of interest since it gives an idea of the surface activation caused by the respective cleaning procedure.

**Figure 4:**
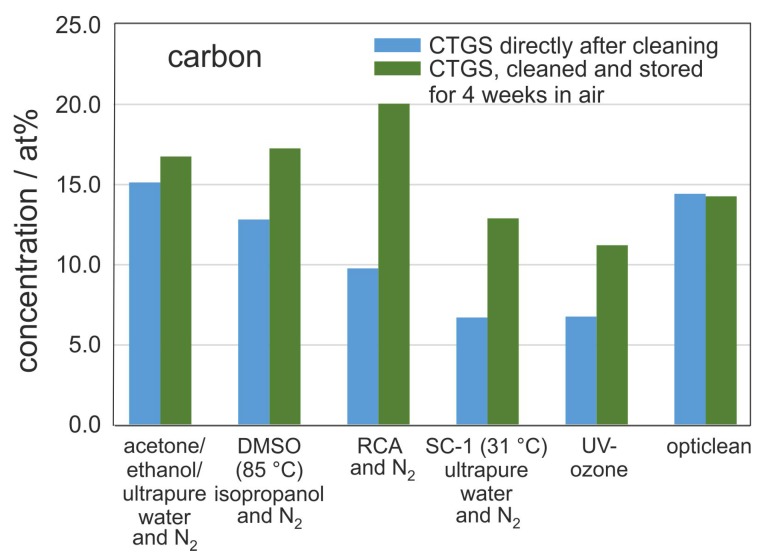
Carbon concentration on the CTGS surface measured for one set of samples directly after the respective cleaning procedure and after 4 weeks of storage under ambient air. Measured with XPS (Al source with neutralizer).

Figure 4 compares the carbon concentration on the surface for one particular sample series directly after cleaning (transferred to XPS system within less than 5 min after cleaning), with the carbon contamination measured after 4 weeks of storage in air. Surprisingly, the carbon content in samples treated with the opticlean procedure did not change during the storage time. Also for the acetone-ethanol-mixture cleaning only a slight increase in carbon content was measured. A possible explanation is that since these two methods showed the highest residual carbon concentration after cleaning, the sample surface might be saturated with C-H so that hardly any further C-H is adsorbed.

It is important to note that the strongest increase in carbon contamination on the CTGS substrates is observed for samples cleaned with RCA procedure. In this case, the carbon concentration doubled from 10 at % C directly after cleaning to 20 at % C after 4 weeks of storage in air, which is the highest value for all samples, indicating a strong activation of the substrate surface for hydrocarbon adsorption. In order to reduce the re-contamination of a silicon substrate surface, Leopold characterized various additives for the RCA procedure [2]. The other cleaning procedures (UV-ozone, SC-1 at 30 ${}^{\circ }$C, DMSO cleaning) showed an intermediate increase of 4–6 at % of carbon.

**Figure 5:**
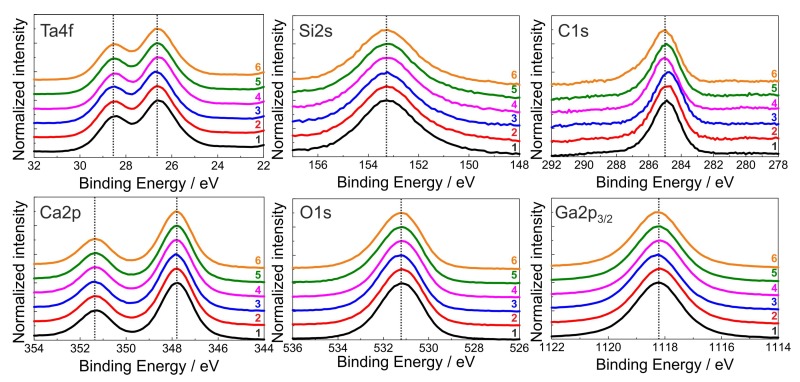
Normalized XPS signals of the elements which form the CTGS substrate (Ca, Ta, Ga, Si, O) and of carbon contamination measured after the various cleaning procedures. The dotted vertical lines serve as guide to visualize the similarity between the measured peaks.

To evaluate the influence of the various cleaning procedures on the surface composition of the CTGS substrate, the XPS spectra of the differently cleaned substrates had to be compared since even slight changes in the composition or in the chemical bonds would have resulted in differently shaped XPS signals. Figure 5 summarizes the XPS signals which are normalized to equal intensity and shifted for better comparison of both the elements of the CTGS substrate and the C concentration caused by contamination. It is seen that there are no differences in the peak shape and no additional side peaks appear. The calculated surface stoichiometry is in acceptable agreement with the nominal one considering the use of standard RSFs and the complicated CTGS chemistry. The small asymmetry of the C1s contamination peaks to higher binding energy points to only minor parts of additional C-O species at around 286.6 eV. Assuming a Gauss-Lorentz peak shape for peak fitting, the amount of this C-O species was always less than 10% in comparison with the main C-H peak (284.8 eV). This clearly proves that the substrate chemistry is not affected by the cleaning procedures. Besides this slight asymmetry, no additional C-X peaks were observed, such as carbonates, which should appear at around 290 eV. Also after the 4 weeks storage period, no additional spectral features such as carbonates were observed.

## 4. Summary and Conclusions

Six different substrate-cleaning procedures for CTGS substrates have been evaluated with respect to the residual contamination by means of XPS measurements. During the procedure of sawing the substrates by processes such as covering with the photoresist, sawing in water, and the final removal of the photoresist, certain amount of contamination might be introduced on the substrate surface. The results of the cleaning processes show significant differences in carbon, sodium, and iron concentrations which were reproducible in separate cleaning series. The UV-ozone procedure has the highest efficiency in the reduction of carbon contamination. Concerning the sodium contamination, the SC-1 cleaning leads to the lowest Na values. The iron content is most successfully reduced by the RCA procedure. Another important result is that the XPS peak shape and also the concentration ratio of the elements of the substrate (Ca, Ta, Ga, Si, O) did not change by cleaning; that is, the chemistry of the substrate surface is not modified by these procedures.

As a final conclusion for the cleaning of CTGS substrate surfaces under ambient conditions, a two-step cleaning procedure consisting of the SC-1 at 30 ${}^{\circ }$C and a subsequent UV-ozone cleaning directly prior to the deposition of the metallization is suggested to achieve the best results with regards to the removal of metal and hydrocarbon contaminations. However, in the case of very critical technological processes which require the lowest level of heavy metal contaminations, the SC-2 cleaning step should also be carried out. In other cases, the SC-1 cleaning at 30 ${}^{\circ }$C will result in very low metallic residues, and the additional UV-ozone procedure minimizes the carbon concentration on the CTGS surface.

To prevent a re-contamination with carbon between the UV-ozone cleaning and the deposition of a film on the substrate, the UV-ozone process should be directly implemented in the deposition chamber or into a directly connected pre-treatment vacuum chamber so as to avoid vacuum interruption between the UV-ozone cleaning and the subsequent film deposition.
